# C-reactive protein as the biomarker of choice to monitor the effects of exercise on inflammation in Parkinson’s disease

**DOI:** 10.3389/fimmu.2023.1178448

**Published:** 2023-05-12

**Authors:** Niyati Mehta, Nijee S. Luthra, Daniel M. Corcos, Giamila Fantuzzi

**Affiliations:** ^1^ Department of Physical Therapy and Human Movement Sciences, Northwestern University, Chicago, IL, United States; ^2^ Movement Disorder and Neuromodulation Center, University of California, San Francisco, San Francisco, CA, United States; ^3^ Department of Kinesiology and Nutrition, College of Applied Health Science, University of Illinois at Chicago, Chicago, IL, United States

**Keywords:** C-reactive protein, Parkinson’s disease, inflammation, exercise, biomarker

## Abstract

Parkinson’s disease (PD), a heterogeneous disease with no disease-modifying treatments available, is the fastest growing neurological disease worldwide. Currently, physical exercise is the most promising treatment to slow disease progression, with evidence suggesting it is neuroprotective in animal models. The onset, progression, and symptom severity of PD are associated with low grade, chronic inflammation which can be quantified by measuring inflammatory biomarkers. In this perspective, we argue that C-reactive protein (CRP) should be used as the primary biomarker for monitoring inflammation and therefore disease progression and severity, particularly in studies examining the impact of an intervention on the signs and symptoms of PD. CRP is the most studied biomarker of inflammation, and it can be detected using relatively well-standardized assays with a wide range of detection, allowing for comparability across studies while generating robust data. An additional advantage of CRP is its ability to detect inflammation irrespective of its origin and specific pathways, an advantageous characteristic when the cause of inflammation remains unknown, such as PD and other chronic, heterogeneous diseases.

## Introduction

Physical exercise is a promising treatment to slow disease progression in various chronic, complex, heterogeneous diseases that share an inflammatory component, such as metabolic syndromes, type II diabetes, multiple sclerosis, Parkinson’s Disease (PD) and many others. Several ongoing intervention studies are assessing the effectiveness of multiple exercise modalities and intensities on disease progression (1–4). In this perspective, we will focus on PD.

PD is the fastest growing neurological disease worldwide and has no disease-modifying treatments. Despite the heterogeneity of its signs and symptoms, treatment of PD relies largely on dopaminergic medications to alleviate motor symptoms. However, over time, the effectiveness of these medications is challenged by continued disease progression and a rise in adverse effects (5, 6). A growing body of evidence supports the beneficial role of exercise in PD. In animal models of PD, exercise is neuroprotective (7) while in humans multiple exercise modalities reduce signs and symptoms of PD (8–10). However, the mechanisms by which exercise provides benefits in PD–as in many other diseases–remain unclear. Inflammation is an emerging component of PD pathogenesis that can be a target for neuroprotection and disease modification (11). Indeed, at least one study reports that chronic use of nonsteroidal anti-inflammatory drugs reduces the risk of PD by about 45%, suggesting that inflammation may play a pathogenic role in PD (12).

Several biomarkers, both individually and as multi-molecular panels, can be used to determine the presence of and quantify the severity of inflammation. Of these, the acute-phase protein C-reactive protein (CRP) is the most studied (13). Here, we elaborate on the reasons for selecting CRP as the biomarker of choice to monitor inflammation in response to exercise in PD and in studies examining the effect of exercise in other diseases with an inflammatory component (14).

## C-reactive protein

Circulating levels of CRP increase rapidly during the acute-phase response, which can be initiated by infection, inflammation, or trauma (15). Inflammatory mediators such as the cytokine IL-6 induce transcription and translation of the CRP gene in hepatocytes (16), with other mediators and cell types also contributing to the rise in circulating levels of CRP (17–19). The biology and regulation of production of CRP have been extensively reviewed and we refer the reader to this vast literature for details (16, 20–24).

The designation of CRP as an acute-phase protein is misleading because levels of CRP (and of other positive acute-phase proteins) increase in virtually all conditions characterized by inflammation, irrespective of whether the course is acute or chronic. In response to an acute infection, CRP levels in peripheral blood can reach concentrations >1 g/L, i.e., thousands of folds up from the ≤1 mg/L observed in non-infected individuals (21). However, CRP levels increase more modestly, yet significantly and consistently, in a wide range of chronic, non-infectious conditions, such as cardiovascular disease (CVD), accelerated vascular aging, autoimmune diseases, obesity, Type 2 Diabetes, Alzheimer’s disease, and PD (14, 20, 25). In these conditions, CRP levels rarely reach the peak observed during acute infections, largely staying below 10 mg/L, but they signal the presence of low grade, chronic inflammation (26). Inflammaging, the presence of low-level chronic inflammation in older adults, is also associated with a modest but consistent elevation in CRP (27).

## CRP in Parkinson’s Disease

Plasma and cerebrospinal fluid (CSF) CRP levels are associated with PD risk, prognosis, and symptom severity. A meta-analysis of 23 studies shows that individuals with PD have significantly higher CRP levels both in the peripheral circulation and in the CSF compared with matched healthy controls (13), indicating that either inflammation is a risk factor for PD or that PD leads to inflammation, and possibly both. Newly diagnosed PD patients have higher systemic CRP levels than people without PD, suggesting that inflammation is already present in the early stages of disease (28). Additionally, across the time course of the disease, patients with PD exhibit higher systemic and CSF CRP levels compared to healthy controls (13) and, independent of disease duration or symptom severity, baseline CRP levels in patients with PD are associated with risk of death and predicted life prognosis (29). CRP levels are also related to PD disease stage, as patients with higher Hoehn & Yahr scores, and therefore more severe motor symptoms, exhibit higher levels of systemic CRP (30, 31). One study found that CSF CRP concentrations correlate with motor symptom severity, measured using the Movement Disorders Society Unified Parkinson’s Disease Rating Scale (MDS-UPDRS) motor examination score (Part III), in male PD patients and with measures of cognitive performance in female patients, suggesting a possible sex dimorphism in CRP as a marker of inflammation in PD and/or in the pathogenic mechanisms of motor versus non-motor symptoms (32). Moreover, CSF CRP levels are higher in patients with PD-related dementia as compared to PD patients without dementia (33) and are also associated with severity of depression, anxiety, and fatigue in PD (33). Thus, despite the heterogeneous nature of PD, CRP–and therefore inflammation–is associated with many of its manifestations in terms of risk, progression, and symptom severity.

## CRP: marker or maker?

There are many ways in which CRP may directly contribute to disease pathogenesis in PD, given its known role in the clearance of necrotic material, recruitment of the complement system, and more (34). However, epidemiological studies indicate that CRP is unlikely to play a major direct role in the pathogenesis of PD, similarly to what has been demonstrated in CVD. Genetic variants in the promoter of the CRP gene that modulate circulating levels of CRP have helped clarify the role of CRP in the pathogenesis of CVD. While elevated levels of CRP consistently predict adverse cardiovascular events, epidemiological studies demonstrated a lack of association between CRP genetic variants and CVD (30). That is, high CRP due to genetic variants without underlying inflammation does not increase the risk of CVD by itself, demonstrating that it is the underlying inflammation that contributes to disease risk, not CRP itself. Similarly, in PD, a large Genome-Wide Association Study failed to identify an association between CRP genetic variants and increased risk of PD (35). These studies indicate that while CRP predicts disease risk and progression, its participation in disease pathogenesis is questionable, at best. Thus, in PD, we should consider CRP as a marker rather than a maker, i.e., as a biomarker that detects the presence of inflammation and quantifies its severity rather than as a direct participant in disease pathogenesis.

## CRP and physical exercise

In clinical studies, CRP is well established as a biomarker to monitor the effects of exercise on inflammation. Indeed, more than 400 randomized controlled trials in various populations and at least 80 systematic reviews or meta-analyses have evaluated the effect of different modalities and intensities of exercise on CRP levels. While there may be a short-lived increase in CRP levels after each exercise bout, since exercise can be an acute stressor, most studies indicate that over time physical exercise lowers CRP levels (7, 36), with aerobic exercise being the most beneficial, especially in older adults (36). Evidence suggests that physical exercise reduces CRP levels following a dose-response relationship, with higher intensity exercise causing a greater reduction in CRP over time compared to lower intensity exercise, and with longer interventions being more efficacious than those of shorter duration (37). Although no studies have yet examined the effect of exercise on CRP levels in PD, exercise, particularly aerobic interventions, counteract the increase in CRP that accompanies aging (27, 38). This is relevant to PD, as age is its primary risk factor (39–41), and can be described as a pre-PD state (39). Furthermore, exercise is a critical component in the prevention and management on Type 2 Diabetes, a condition that is associated with more severe symptoms and accelerated progression of PD and that shares inflammation as a pathogenic mechanism (14).

## Discussion

There are several ongoing trials examining the effects of exercise interventions on PD (2, 4), including the Study in Parkinson’s disease of exercise phase 3 (SPARX3). SPARX3 is a Phase 3, multisite, randomized, two-arm (1:1 allocation), parallel group, evaluator-blinded, clinical trial to test the superiority hypothesis that high-intensity, endurance treadmill exercise slows the progression of the signs of PD compared to moderate-intensity endurance treadmill exercise (4). A change in the MDS-UPDRS Part III score is the primary outcome. Several biomarkers serve as secondary outcomes that might point to the mechanisms underlying the effects of exercise intensity in PD, including a potential reduction in inflammation (42).

In SPARX3, we could have chosen a variety of biomarkers to monitor inflammation in response to endurance exercise. Indeed, we plan to explore levels of cytokines and several other mediators in participants’ systemic circulation. However, several reasons led us to select CRP as the sole inflammation-related pre-specified outcome.

The fact that CRP is by far the most studied biomarker of inflammation, both in exercise and in PD as well as in many other diseases, will permit comparison between the findings of SPARX3 and those of hundreds of other studies. Moreover, compared to other mediators, CRP allows for better comparability across studies due to the higher standardization of the CRP assay compared to that of most cytokines and many other inflammatory mediators. Moreover, unlike most cytokines, levels of CRP can be reliably quantified even in the absence of inflammation, thus avoiding the clustering of values at the lower edge of the sensitivity curve that plagues most cytokine assays.

Lastly, and perhaps most importantly, CRP is nonspecific, meaning that it can detect the presence of inflammation, and quantify its changes, irrespective of the ultimate origin of the inflammatory response and of the mechanisms at play. This characteristic of CRP is very useful in PD, in which the cause and pathways of inflammation have not been identified. Similarly, because the mechanisms by which exercise reduces inflammation are also unidentified thus far, the lack of specificity of CRP becomes an asset. Studies that aim to investigate the location, triggers, and pathways of inflammation in PD, and the mechanisms by which exercise reduces inflammation, must quantify specific markers, such as individual cytokines. However, if the aim of a study is to determine whether inflammation is present and/or whether its severity can be altered through an intervention–as is the case in SPARX3–a nonspecific marker such as CRP is more appropriate, as its modulation is independent of the origin and characteristics of the inflammatory response. Thus, nonspecific is not always a dirty word, particularly when it comes to the interplay of PD, exercise, and inflammation. If SPARX3 demonstrates that endurance exercise is associated with reduced levels of CRP, as is our hypothesis, studies evaluating the mechanisms underlying this effect will be warranted.

For all the reasons outlined above, CRP should be utilized as the biomarker of choice for evaluating the response to exercise interventions in PD and other neurodegenerative diseases and chronic conditions. However, like any biomarker, there are limitations in the utility of CRP. First, CRP is sensitive to any form of inflammation, meaning that inflammation unrelated to PD, such as an infection, will increase CRP levels. This, however, is a challenge of nearly all inflammatory biomarkers, and not unique to CRP. Also, CRP levels are affected by genetics, and this will influence CRP levels irrespective of disease severity and exercise effectiveness. However, this can be overcome by averaging CRP levels across subjects to reduce the effect of genetic variations on CRP levels and/or by utilizing intra-subject longitudinal comparisons. Despite these limitations, we argue that CRP is the most effective biomarker for monitoring the effects of exercise interventions on the level of inflammation in PD and other conditions.

## Conclusion

Researchers investigating the effects of physical exercise in PD and many other diseases are faced with lack of knowledge about the specific pathways in which inflammation is implicated both in disease pathogenesis and in the beneficial effects of the intervention. Here we argued that the current situation should not hamper progress in the field, that evaluating the effectiveness of exercise in PD and other conditions does not need to wait for mechanistic studies to elucidate such pathways. Indeed, choosing CRP as the biomarker of choice to monitor the state of inflammation during an intervention overcomes many of the limitations of current knowledge.

## Data availability statement

The original contributions presented in the study are included in the article/supplementary material. Further inquiries can be directed to the corresponding author.

## Author contributions

NM – execution, writing, and editing of the final version of the manuscript. NL – writing and editing of the final version of the manuscript. DC – writing and editing of the final version of the manuscript. GF – execution, writing, and editing of the final version of the manuscript. All authors contributed to the article and approved the submitted version. 
